# Development and Initial Evaluation of Specific Immersive Competence in Virtual Reality–Based Medical Assessments: Exploratory Observational Study

**DOI:** 10.2196/82136

**Published:** 2026-04-08

**Authors:** Verena Schreiner, Joy Backhaus, Marco Lindner, Melina Heinisch, Sarah König, Sebastian Oberdörfer, Tobias Mühling

**Affiliations:** 1 Institute of Medical Teaching and Medical Education Research University Hospital Würzburg Würzburg Germany; 2 Chair for Human-Computer Interaction Julius Maximilians University of Würzburg Würzburg Germany

**Keywords:** virtual reality, immersive competence, OSCE, objective structured clinical examinations, medical education, performance assessment, exploratory study, pilot study

## Abstract

**Background:**

Virtual reality (VR) is increasingly used in medical education for training and examination purposes; yet, learners’ performance in VR-based assessments may be influenced by more than clinical competence alone. Immersive competence (IC) has been proposed as a relevant factor in VR-based performance assessment. While general IC captures application-independent VR interaction skills, domain-specific applications may require an additional construct, specific immersive competence (specific IC), reflecting context-dependent interaction proficiency.

**Objective:**

This study aimed to develop and examine the initial psychometric characteristics of a newly developed in situ checklist for assessing specific IC, to explore the relationship between specific and general IC as well as related human abilities and characteristics, and to capture preliminary associations between both IC measures and medical performance in a VR-based objective structured clinical examination (OSCE) station.

**Methods:**

In this observational follow-up study, 21 final-year medical students who had previously completed a curricular OSCE including a VR-based emergency medicine station were recruited. General IC was assessed using the VR competence app, and specific IC using a checklist embedded in the original VR simulation. Additional measures included self-reported technological affinity, spatial ability, and OSCE performance scores. Analyses focused on descriptive statistics and exploratory associations, including item difficulty, item-total correlations, and internal consistency for the specific IC checklist. Pearson *r* was used to examine associations among variables.

**Results:**

The final 13-item specific IC checklist demonstrated acceptable internal consistency for an early-stage instrument (Cronbach α=0.79) and balanced item difficulty (mean P 0.56, SD 0.28). Specific IC showed a strong exploratory association with general IC (*r*=0.56; *P*=.008) and with prior 3D application experience (*r*=0.57; *P*=.007), but no relevant association with spatial ability. Both general and specific IC showed borderline, moderate associations with VR-OSCE performance (*r*=0.41; *P*=.06 and *r*=0.37; *P*=.09, respectively), while neither was related to overall analog OSCE performance.

**Conclusions:**

In this pilot sample, specific IC emerged as a psychometrically accessible construct aimed to capture context-sensitive VR interaction skills. The proposed in situ approach offers a feasible and scalable method to assess this construct within domain-specific VR applications. Although associations with medical performance were exploratory and limited by sample size, the findings suggest the relevance of IC as a potential source of construct-irrelevant variance in VR-based assessments and support further investigation in larger, confirmatory studies.

## Introduction

Virtual reality (VR) is increasingly being used in medical education, providing students with immersive and interactive environments that support skill acquisition, deliberate practice, and feedback-driven learning [1]. VR-based training formats can support the development of both technical skills, such as those required in laparoscopic surgery [2], as well as procedural competencies, including clinical reasoning [3], teamwork [4], and the management of rarely encountered scenarios such as medical emergencies [5] or brain death diagnostics [6]. Beyond its role in simulation-based training, VR is progressively being integrated into performance assessment, offering authentic and interactive scenarios for the structured evaluation of clinical competencies [7-9]. A growing number of studies have explored the use of VR for high-stakes evaluations, such as virtual objective structured clinical examinations (OSCEs), in which learners’ clinical decisions and actions are assessed within predefined clinical scenarios in an immersive environment [10-13]. Such formats can facilitate precise logging of user behavior, consistent scenario delivery across candidates, and automated scoring options. Moreover, VR-based assessments suggest advantages in terms of scalability, objectivity, and ability to replicate complex clinical environments that are challenging or resource-intensive to recreate in conventional OSCE settings [8,10-13].

As the boundaries between learning and testing environments become increasingly fluid, the question emerges as to whether learners' performance in VR accurately reflects their actual clinical competence, or whether it is confounded by interface-related factors. This concern is reinforced by the fact that VR users must adapt to abstract and often nonstandardized metaphorical representations [14], such as administering medication via 2D menus or receiving feedback through controller vibrations. Even more recent approaches using hand-tracking rely on abstracted gestures that must be learned and mastered by the user [11]. Steed et al [15] introduced the concept of immersive competence (IC), defined as a user’s familiarity and skill with immersive controls. IC thus represents the user readiness required for interaction tasks in VR applications, the absence of which can lead to difficulties with controls, interactions, or navigation within the VR environment. IC increases with prior VR experience [16,17] but also appears to be trainable [18], thereby reducing cognitive load and enhancing multitasking performance in VR environments [18]. From a validity perspective, IC may constitute a source of construct-irrelevant variance [19], as it can influence performance in VR-based assessments without being part of the intended construct of clinical competence. Indeed, marked differences in item discrimination parameters between VR-based and content-equivalent analog OSCE stations already suggested a systematic bias in VR-based examinations, potentially driven by differences in IC [12]. To capture the construct of IC, based on previous approaches [20,21], a novel assessment tool was developed: the VR competence app [17]. This application presents users with a range of prototypical interactions commonly required in immersive environments, such as picking up and placing objects, spatial navigation, and the activation of user interface (UI) elements. Importantly, it is content-agnostic and does not reference any specific educational or clinical scenario. As such, it aims to provide a standardized and domain-independent estimation of a user’s general interactional proficiency in VR (general IC).

However, VR interfaces vary considerably across applications and hardware platforms [22]. This heterogeneity can be confusing for users and hinder the effective implementation of this technology [23,24]. Differences include customized controller mappings, unique interface layouts, and spatial or logistical features specific to a simulation [22,25]. For instance, general familiarity with UI activation in VR environments does not necessarily translate into proficiency with context-specific tasks such as adjusting infusion rates or navigating electronic menus in a VR-based emergency room simulation. The importance of distinguishing between general (medium-related) and specific (content- or task-related) operational competences has been highlighted by studies from other fields: For example, general internet skills like navigating websites or conducting basic research have been shown to be a poor predictor of more specific internet skills such as selecting appropriate websites for information retrieval or critically evaluating information sources [26]. Similarly, principles derived from the acquisition of simple motor tasks often fail to generalize to complex, context-rich skill environments [27]. While usability measures focus on the system’s design quality [28] and general IC captures abstract interaction skills [17], neither adequately reflects a user’s ability to operate a specific VR interface under real-world constraints. Technological and digital literacy are typically self-reported and too general to capture the specific, context-bound interaction skills required in VR-based assessments [29,30]. This underscores the need for a complementary measure that reflects application-specific motor, cognitive, and interface-related skills. Here, we introduce the concept of Specific Immersive Competence (specific IC)—a context-sensitive construct that reflects a user's ability to perform interactional tasks within a particular, domain-relevant VR application. Specific IC may ideally be measured directly in situ*,* that is, directly within the target application, for example, by using guided, nonclinical tasks designed to isolate interactional skills from domain-specific knowledge. This approach aims to enhance contextual congruence between training, task, and testing environments, in line with best practices in simulation-based assessment [31,32]. In the implementation presented here, users are guided through tasks composed of exemplary interaction steps (eg, connecting an IV line or confirming a medication dose via UI). Task completion within a given time frame is logged using an objective checklist format. This enables a targeted assessment of users’ interactional readiness for high-stakes VR-based training and assessments.

To explore the relationship between specific and general IC and their association with students’ performance, as well as to gather initial validity evidence for the specific IC construct, we conducted a follow-up study within an existing curricular assessment framework. Specifically, we recruited participants from a cohort of advanced clinical-phase medical students who had previously completed a VR-based station as part of their OSCE [12]. This post hoc recruitment strategy allowed us to link newly collected data on both general and specific IC with previously obtained medical performance scores—both for the VR station and for the OSCE as a whole. In doing so, this pilot study pursued three objectives: (1) we first assessed the initial psychometric characteristics of the newly developed in situ checklist for assessing specific IC, including item difficulty, discrimination (via item-total correlations), and internal consistency; (2) second, we explored patterns of association between specific and general IC as well as established human abilities and characteristics to explore whether both measures capture potentially distinct facets of VR interaction competence; and (3) finally, we analyzed the preliminary associations between both IC measures and students’ medical performance in a VR-based OSCE station as well as the overall OSCE score, to provide initial evidence of IC’s potential impact on VR-based performance assessment.

## Methods

### Apparatus

The VR competence app (developed by the Chair for Human-Computer-Interaction, University of Wuerzburg, Germany) was used for the assessment of general IC. STEP-VR (Simulation-based Training of Medical Emergencies for Physicians Using Virtual Reality; version 0.11b; ThreeDee GmbH) was used as a VR simulation of complex emergencies for in situ assessment of specific IC [33]. The application places users in a virtual emergency department, where they are required to diagnose and treat patients using a variety of medical procedures. STEP-VR is a joint development between a 3D visualization company and the University Hospital of Würzburg and had previously been used for the VR-OSCE station [12].

The hardware setup consisted of a Schenker XMG Core 15 Laptop (Schenker Technologies GmbH; chipset: Intel Core i7-9750H, 6×2.6 GHz; graphics adapter: Nvidia GeForce GTX 1650, 4 GB GDDR6 VRAM) and a Meta Quest III VR head-mounted display. Corresponding Meta Quest III controllers were used, which provide standard buttons, thumbsticks, and haptic feedback.

### Study Design

This exploratory observational follow-up study was designed to develop and operationalize the construct of specific IC and to generate preliminary psychometric and correlational evidence. The study was conducted at the skills lab of a German medical school with a 6-year medicine degree curriculum between July 2024 and May 2025. Participants were retrospectively recruited from a cohort of advanced clinical-phase medical students who had completed a curricular OSCE in July 2023, including a VR-based emergency medicine station (VR-OSCE station). Recruitment was through semester mailing lists. The study appointments took place outside regular teaching hours in the form of individual sessions. To examine the relationship between general and specific IC and students’ medical performance, we performed individual post hoc sessions with students who had previously completed the VR-OSCE station. As the study aimed to recruit the complete eligible cohort of students who had previously participated in the VR-based OSCE, a formal a priori sample size calculation was not performed, as it was determined by participant availability. However, at the time of the study, participants were in their final year of medical training and thus dispersed across various clinical rotations and institutions, making recontact and scheduling particularly challenging. From the original cohort of 57 students, a total of 21 (37%) could be recruited for the follow-up. As in the original VR-OSCE study, no a priori exclusion criteria were applied, and all enrolled participants completed the study protocol in full.

**Figure 1:**
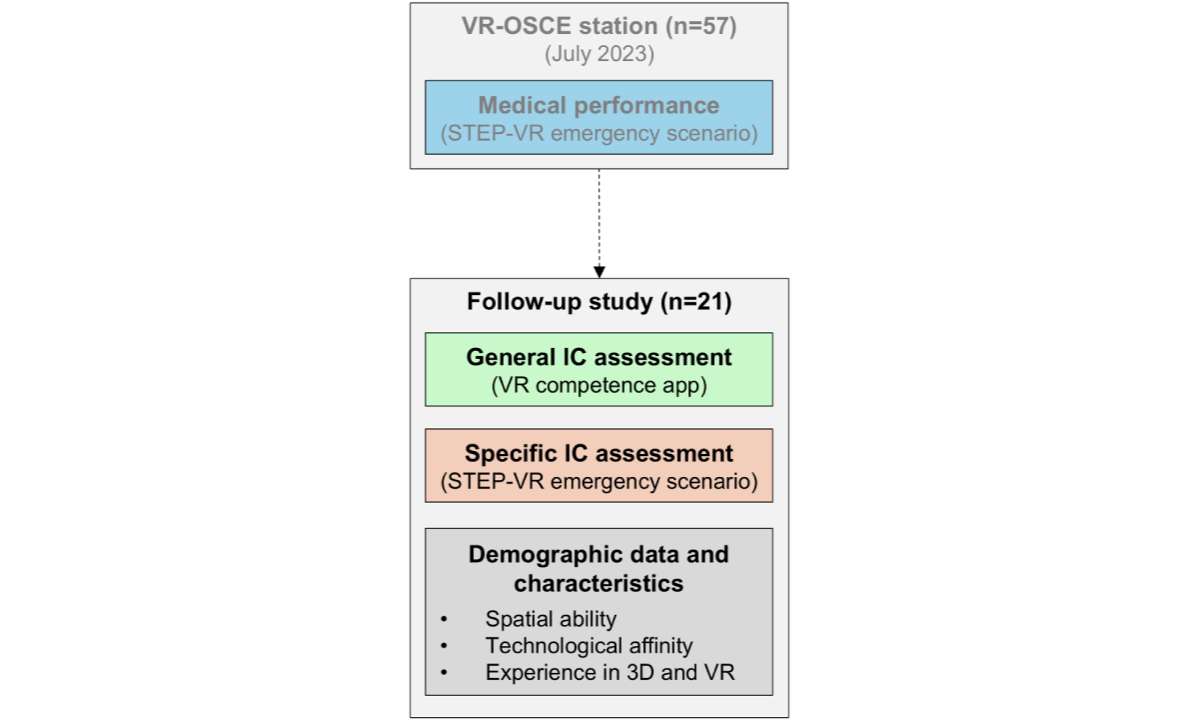
Study Procedure and Data Collection. This study was conducted with participants who completed the virtual reality–objective structured clinical examination station in July 2023. IC: immersive competence; OSCE: objective structured clinical examination; VR: virtual reality.

Each session lasted approximately 30 minutes. After the study procedure had been explained and informed consent was given, participants received a short basic introduction to the operation of the VR headset and controllers. Following, general IC was assessed using the VR competence app (10 minutes). After an organizational break of approximately 2 minutes, the specific IC was tested using the domain-specific application (6 minutes), which was already used during the original VR-OSCE station. At last, participants completed a questionnaire assessing demographic data (including age, gender, and cumulative experience with 3D and VR applications excluding the previously conducted VR-OSCE), technological affinity, and spatial ability (10 minutes). Figure 1 illustrates the study procedure and data collection.

### General IC Assessment

**Figure 2:**
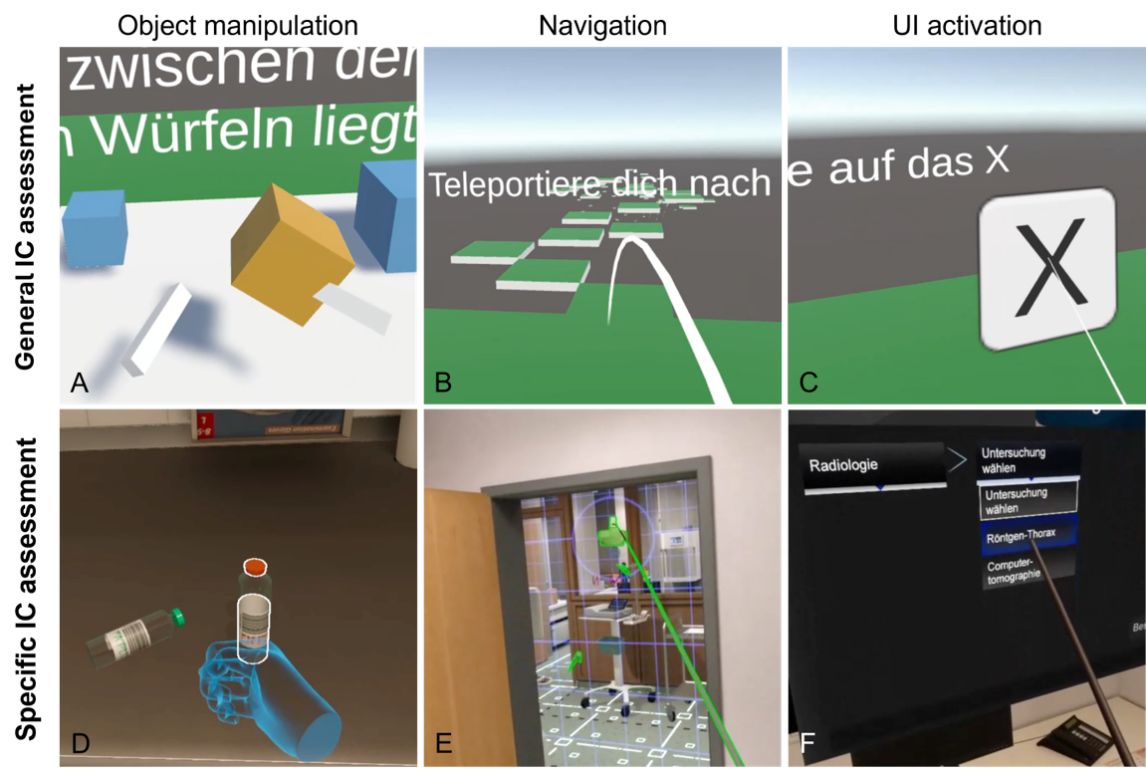
Exemplary interaction steps illustrating general and specific immersive competence domains across different levels of abstraction. For each domain, abstract interaction tasks from the general immersive competence assessment (virtual reality competence app) are displayed above their concrete, domain-specific implementation within STEP-VR (virtual emergency department). A/D: Object manipulation (specific immersive competence: picking up blood culture bottles). B/E: Navigation (specific immersive competence: teleporting to the nurse station). C/F: User interface activation (specific immersive competence: selecting diagnostics and consults in the virtual computer menu). IC: immersive competence; UI: user interface.

General IC was measured essentially as previously described [17] using the VR competence app. This app comprises 8 standardized challenges reflecting common VR interactions from the categories of object manipulation, spatial navigation, and UI activation. For 1 minute, the user is asked to complete as many repetitions of the respective interaction as possible while task difficulty increases. Notably, this application is content-agnostic and independent of any specific educational or clinical scenario. Each task was scored automatically and summed into a total IC score. A visual example is provided in Figure 2.

### Specific IC Assessment

Specific IC was assessed using a checklist-based performance measure embedded within the VR emergency room scenario previously used for summative OSCE assessment. This approach ensured high ecological validity and close alignment between IC measurement and the application-specific interaction demands encountered in high-stakes VR-based settings. To isolate immersive competence from medical knowledge, all tasks were deliberately designed to be independent of clinical reasoning. Participants received standardized verbal instructions (eg, “Measure oxygen saturation”), which were decomposed into discrete, technically feasible interaction steps (eg, “Pick up and attach SpO₂ clip” and “Read out SpO₂ value”). Each step was classified as object manipulation, spatial navigation, or UI activation, in accordance with established VR interaction typologies [16] (Figure 2). Steps involving more than one category were assigned to all relevant categories for analytical purposes.

Initially, a pool of 20 interaction steps was defined based on expert walkthroughs of the VR scenario and a systematic mapping to the application’s core control logic. Steps were selected to reflect a range of difficulty levels and to ensure coverage of all three interaction categories. This initial item pool (Multimedia Appendix 1) was reviewed by one VR developer (SO), one clinician with specialized qualification in medical education (SK), and one clinician experienced in simulation-based training (TM) to confirm technical feasibility and independence from medical knowledge. During pilot testing with five medical students recruited from the skills lab tutor pool (seventh to ninth semester, without specific prior VR experience and not part of the VR OSCE cohort), 6 steps were consistently completed by all participants and thus showed no variance. These were excluded from further analysis, as they lacked discrimination. One additional item (selecting “Surgical Consult”) showed a negative item-total correlation (*r*=–0.06) in our data, likely due to a software usability issue in menu navigation that was identified retrospectively and was therefore removed. The final IC checklist included 13 dichotomously scored steps (0=incorrect or incomplete; 1=correctly completed within time limit), with a maximum raw score of 13 points (Table 1). Time limits for each step were calibrated based on internal pilot runs to reflect escalating difficulty, ensuring that early steps were accessible for users with low IC, while later steps were aimed to challenge higher proficiency levels.

**Table 1:** Tasks, instructions, and individual interaction steps with their corresponding time limits, assigned interaction categories.

Task	Instruction	Time (s)	Step	Interaction step	Interaction category	P^a^	r’^b^
Measuring SpO_2_^c^	Take the SpO_2_ clip from the desk and attach it to the patient`s right hand. Then read the SpO_2_ value from the vital signs monitor aloud.	10					
			1	Attach SpO_2_ clip to patient	O^d^, N^e^	0.71	0.14
			2	Read out SpO_2_ value	U^f^	0.52	0.31
Administering saline	Take an IV catheter out of the box in the labelled drawer and place it on the patient`s arm. Then pick up a 1 liter IV fluid bag from the drawer and hang it on the IV pole. Increase the rate of the infusion to 1000 ml per hour.	30					
			3	Pick up IV^g^ catheter	O, N	0.90	0.29
			4	Pick up IV fluid bag	O, N	0.90	0.22
			5	Set desired infusion rate	U	0.48	0.48
Taking blood cultures	Take a blood culture bottle from the “Microbiology” drawer and connect it to a cannula. Use this blood culture bottle to draw blood from one of the patient’s arms. Then place it on the desk.	15					
			6	Pick up blood culture bottle	O, N	0.90	0.14
			7	Connect bottles to cannula	O	0.57	0.48
			8	Draw blood	O	0.43	0.54
			9	Place filled bottles on desk	N	0.05	0.23
Administering oral medication	Take a box of clopidogrel from the “oral medication” drawer. Drop the medication onto the patient’s mouth.	10					
			10	Pick up oral medication	O, N	0.86	0.22
			11	Drop medication on patient’s mouth	N	0.48	0.55
Administering IV medication	Take a box of aspirin from the “IV medication” drawer. Fill it into a 50ml bottle and confirm the dose. Then hang the infusion on the IV pole.	15					
			12	Prepare IV push and confirm dose	U	0.33	0.59
			13	Hang infusion on IV pole	O, N	0.19	0.60

^a^P: resulting difficulty; mean 0.56, SD 0.28.

^b^r’:discrimination values; mean 0.37, SD 0.17.

^c^SpO_2_: peripheral capillary oxygen saturation.

^d^O: object manipulation.

^e^N: navigation.

^f^U: user interface activation.

^g^IV: intravenous.

### Technological Affinity

Participants’ attitudes toward and confidence with technology were assessed using 2 subscales of the validated German Affinity for Technology- Electronic Gadgets (TA-EG) questionnaire [30], namely “enthusiasm” (eg, “I enjoy trying out new technical devices”) and “competence” (eg, “I am confident using complex devices without help”). Each scale comprised 5 items rated on a 5-point Likert scale (1=strongly disagree to 5=strongly agree). Scores were averaged separately for each scale.

### Spatial Ability

Spatial ability was assessed via a German version of a paper-based mental rotation task used in psychometric aptitude testing [34] following a similar principle as the original version [35]. Participants were shown 10 3D tube-shaped figures and asked to identify matching rotated versions from four options. Each correct response was awarded one point; thus, scores ranged from 0 to 10.

### Medical Performance

Students’ medical performance data were extracted from the original OSCE scores [12]. Two performance indicators were analyzed: The VR-OSCE station score (range 0-100%) and the overall OSCE score (calculated as the mean of all 9 other OSCE stations, range 0-100%).

### Statistical Analysis

Given the exploratory nature and limited sample size, analyses focused on descriptive statistics, effect size patterns, and correlation coefficients rather than confirmatory hypothesis testing. Mean (SDs) and difficulties (P) as well as item discrimination (r’), were used to characterize the sample and items of the specific IC assessment. Given the small sample size, which precluded common factor analysis, we followed suggestions to enhance the validation of education instruments [36]. Item discrimination (r’) representing item-total correlation for the dichotomous items of the specific IC assessment was calculated using point-biserial correlations.

Normality of data distributions was initially assessed using the D’Agostino–Pearson test, which did not indicate relevant deviations from normality. Accordingly, Pearson correlation coefficients are reported. In response to reviewer feedback, additional sensitivity analyses using Spearman rank correlations were conducted to assess the robustness of the findings against potential violations of normality assumptions.

Point-biserial correlations were calculated in R (version 4.3.1; RRID:SCR_001905; R Core Team), and all other statistical analyses were conducted in GraphPad Prism (Version 10.5.0, RRID:SCR_002798; GraphPad Software Inc). A significance level of α=.05 was used throughout.

### Ethical Considerations

#### Ethics Approval

The local institutional review and ethics board (Ethikkommission der Universität Würzburg) judged the project as not to constitute medical or epidemiological research on human participants and as such adopted a simplified assessment protocol. The project was approved without any reservation under the proposal number 20240208-01.

#### Transparency Statement

The authors confirm that all measures, conditions, and data exclusions associated with this study have been fully reported in the manuscript and the supplementary data (Multimedia Appendices 1-Multimedia Appendices 3). No additional experimental conditions or outcome measures were collected, but not reported.

#### Informed Consent and Compensation Details

Students were informed about the study, and their participation was voluntary. Informed consent was obtained from all participants, who were also provided with information on data processing for the analysis and the publication of results. Contact details were supplied for participants wishing to withdraw their consent to data processing. The decision to participate or not had no consequences on the students’ academic progress. A €30 (US $33.96) book voucher was given to the participants at the end of the study appointment.

#### Privacy and Confidentiality

Data were stored in pseudonymized form on university servers with restricted access, and no personal identifiers were retained beyond the matching of OSCE ID codes. Survey data from the questionnaires were collected anonymously using the EvaSys platform (Evasys GmbH). Data were processed and stored in accordance with local data protection laws.

## Results

### Descriptive Statistics

The final sample included 21 medical students (age: mean 26.5, SD 2.6 years), with 10 identifying as female and 11 as male. Both exposure to 3D applications and prior experience with VR applications apart from the previously conducted VR OSCE were very limited (Table 2). No symptoms of simulation sickness were reported, and all participants were able to complete the study session in full. In reference to the scale midpoint (=3), participants reported moderate levels of technology enthusiasm (mean 2.38, SD 0.87) and above-average self-rated technology competence (mean 3.46, SD 0.68, both on a 5-point Likert scale) on the TA-EG. Spatial ability, measured via a standardized mental rotation test, showed a mean score of 5.48 (SD 2.29) out of 10. General immersive competence, assessed via the VR competence app, yielded a mean performance score of 57.27 (SD 7.38), while specific immersive competence, measured in the VR emergency room simulation, showed a mean of 7.33 (SD 2.62) out of 13. Medical task performance among participants in the original VR OSCE station averaged at 67.73% (SD 14.62), and the overall OSCE score across all stations was mean 80.30% (SD 4.83).

**Table 2:** Participant characteristics, including age, gender, and experience with 3D and virtual reality applications (total n=21).

Characteristics		Values
Age (years), mean (SD)		26.5 (2.6)
**Sex, n (%)**		
	Female	10 (48)
	Male	11 (52)
	Diverse	0 (0)
**Frequency of using desktop 3D-applications, n (%)**		
	Never	14 (67)
	Rarely (<1/month)	4 (19)
	Sometimes (<1/week)	3 (14)
	Frequently (>1/week)	0 (0)
	Daily	0 (0)
**Cumulative experience with VR** ^a^ **applications, n (%)**		
	None	4 (19)
	0-1 hours	11 (52)
	1-10 hours	6 (29)
	10-50 hours	0 (0)
	>50 hours	0 (0)

^a^VR: virtual reality.

### Performance Checklist and Psychometric Properties

Overall, item completion rates ranged from 0.05 to 0.90 (mean 0.56, SD 0.28), indicating a broad range of item difficulty (P). While several basic object manipulation tasks (eg, picking up the oxygen saturation clip or retrieving an infusion bottle) were completed by nearly all participants, more complex UI-dependent steps, such as setting the exact infusion rate, showed substantially lower success rates. Discrimination ranged from 0.14 to 0.60 (Mean 0.37, SD 0.17), with a subset of UI-related and navigation-heavy tasks displaying particularly good discrimination. In contrast, items with extreme difficulty (either too easy or too hard) showed limited ability to differentiate between participants. All performance data are displayed in Table 1.

The internal consistency across all 13 items, calculated using Cronbach's alpha, was α=0.79, suggesting good reliability for an early-stage checklist instrument. Removing low-discrimination items only slightly improved internal consistency (maximum α=0.80), supporting the checklist’s robustness in its current form. For interaction categories Cronbach alpha was α=0.60 (n=8) for navigation, α=0.64 for object manipulation (n=8), and α=0.65 for UI activation (n=3). Solely for navigation removal of the item #1 “Attach SpO_2_ clip to patient” would markedly improve reliability to 0.64.

### Correlation Analyses

Specific IC showed a strong correlation with general IC (*r*=0.56; *P*=.008), providing preliminary evidence for their conceptual relatedness. In contrast, correlations between general and specific IC within corresponding interaction categories were low and nonsignificant for object manipulation (*r*=0.17; *P*=.50) and navigation (*r*=0.17; *P*=.14). The highest correspondence was observed for UI activation (*r*=0.41; *P*=.07).

Moreover, specific IC correlated moderately to strongly with prior 3D experience (*r*=0.57; *P*=.007) and self-rated technical competence (*r*=0.46; *P*=.04). While both general and specific IC were moderately associated with performance in the VR-OSCE station at a borderline level of significance (*r*=0.41; *P*=.06 and *r*=0.37; *P*=.09, respectively), neither correlated meaningfully with overall OSCE performance (*r*=0.16; *P*=.48 and *r*=0.02; *P*=.92, respectively). Similarly, no relevant correlations were found between specific IC and technology enthusiasm or spatial ability.

When decomposing specific IC into interaction categories, object manipulation correlated most strongly with the total specific IC score (*r*=0.94, *P*<.001). Object manipulation and—most notably—UI activation also showed preliminary associations with 3D experience (*r*=0.51; *P*=.02 and *r*=0.67; *P*=.001) and technical competence (*r*=0.54; *P*=.01 and *r*=0.35; *P*=.12).

**Figure 3:**
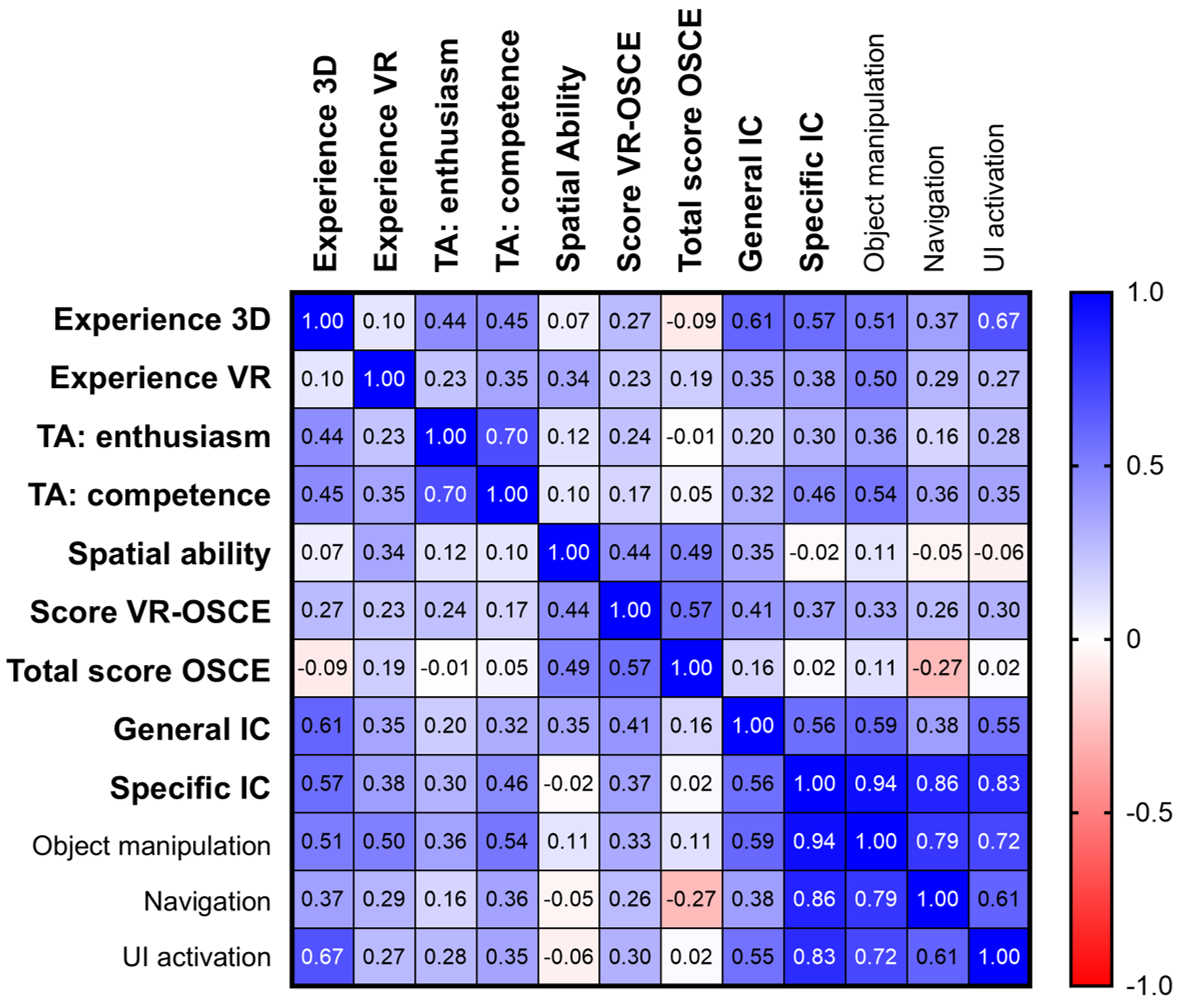
Correlation matrix between prior experience in 3D and virtual reality applications, technical affinity, spatial ability, and students’ performance scores of the virtual reality-objective structured clinical examination station and the total objective structured clinical examination as well as general and specific immersive competence (the latter subdivided into object manipulation, navigation and user interface activation). The heatmap scale (right panel) indicates the color coding for positive (blue) and negative (red) correlations. The more intense the color, the higher the correlation coefficient. Corresponding Pearson significance values are listed in Multimedia Appendix 2. IC: immersive competence, OSCE: objective structured clinical examination, TA: technical affinity questionnaire, UI: user interface, VR: virtual reality.

All Pearson correlations are listed in Figure 3, and their significance values are listed in Multimedia Appendix 2. Importantly, Pearson and Spearman correlation analyses yielded comparable results in terms of direction and magnitude across all key variables, indicating that the reported associations are robust to potential violations of normality assumptions. All Spearman analyses, along with their significance values, are also reported in Multimedia Appendix 2.

## Discussion

### Principal Findings

This pilot study introduces and empirically examines the construct of specific IC as a context-sensitive dimension of VR user readiness, reflecting the ability to successfully perform application-specific interaction tasks independent of domain knowledge. Moreover, it provides initial data to inform further exploration of its potential role in predicting performance in VR-based medical assessments. Building on prior theoretical definitions of IC [15] and on established approaches for assessing broad, application-independent VR interaction skills such as object manipulation, spatial navigation, and UI activation [17,20,21], we argue that domain-specific VR systems may demand an additional, situationally anchored level of interaction proficiency. In response, we evaluated a simple, objective, and reproducible approach for measuring this construct, referred to here as “specific IC,” directly within a domain-specific VR application.

Our first objective addressed the initial psychometric evaluation of our new method for measuring specific IC. Within the limited sample of this study, the developed checklist demonstrated satisfactory psychometric properties for an experimental prototype: overall internal consistency was acceptable (Cronbach α=0.79), meeting common thresholds for exploratory research, where α values above 0.70 are typically considered adequate for group-level comparisons [37]. However, internal consistency for subscales measured by Cronbach α requires further exploration. Since the α coefficient is based solely on interitem correlation, it is possible that lower values were caused by subgroups, which could not be investigated due to the small sample. Overall difficulty levels were balanced and largely within the desired range, which is typically between 0.4 and 0.8 [38]. Most items demonstrated good item-total correlations (>0.3), indicating that they were able to differentiate well between participants with varying levels of specific IC. An item discrimination above 0.3 is usually considered indicative of a useful item, especially in early test development [38]. One exception was item #6, which displayed poor discrimination and an item difficulty of 0.90. Since our small sample size did not allow conclusions about the specific IC range of the general population, we retained this item to avoid potential floor effects in future studies with different populations. Future refinements may benefit from larger, more representative samples and from user interviews, for example, to identify subtle misunderstandings of task instructions.

Our second research objective was to explore its relationship with general IC and related human abilities to determine whether the 2 constructs reflect distinct aspects of VR readiness. We observed a moderate-to-strong and significant correlation between general and specific IC scores, while their subscales did not correlate. This suggests that while both constructs may partially share underlying cognitive resources, they also reflect distinct aspects of VR user readiness, similar to findings from other domains [26,27]. Beyond the limited evidence of this small correlational study (which may not have the statistical power to effectively detect smaller correlations), future studies could further investigate the distinctness of general and specific IC using targeted training interventions to manipulate each construct independently, as well as latent-variable approaches (eg, confirmatory factor analysis).

Both general and specific IC showed significant preliminary associations with participants’ prior exposure to 3D applications, which is consistent with previous findings on (general) IC that describe these abilities as (formally or informally) trainable [16-18]. At first glance, this may raise the question of whether IC represents a redundant construct. However, due to the heterogeneity of VR experiences (eg, gaming vs educational use, passive vs interactive applications), as well as differences in preexisting motor and perceptual abilities, two individuals with similar amounts of VR experience may differ substantially in their IC. This individual variability and empirical decoupling of IC from mere experience is further supported by the multidimensional structure of IC, with separable interaction domains showing heterogeneous associations with prior experience and only moderate correlations with VR and 3D exposure in this study. Moreover, unlike experience, IC constitutes a target parameter that can be systematically assessed, compared across users, and potentially trained to enable fair conditions for immersive assessments. While familiarization or training in VR is likely to increase immersive competence on average, the extent of this effect, as well as the contributions of prior VR experience or preexisting motor and perceptual abilities, remains unclear without explicit measurement of IC. This underscores the need to assess IC directly rather than assuming VR user readiness based on exposure alone. The comparatively stronger effect of 3D experience over prior VR exposure in this study is likely due to the limited variance in the latter, as most participants had minimal VR experience, thereby reducing its explanatory power. Importantly, reported *P* values should be interpreted cautiously in light of the study’s limited statistical power.

While there is some conceptual overlap with constructs such as self-reported technological competence, correlations with these measures remained weak and nonsignificant - both in this and in previous studies [17]. In our sample, participants’ self-rated technology competence was slightly above average compared with normative data from a large German validation study of the TA-EG [30], whereas enthusiasm was somewhat lower, providing additional context for the observed weak associations. The absence of a significant correlation with spatial ability in the present work is noteworthy, especially given that similar—although not identical—mental rotation tasks were used in the earlier study [17]. One possible explanation could be the different disciplinary background of the present sample and participants’ potential familiarity with the mental rotation test, which, for some students, forms part of the medical school aptitude test, potentially leading to biased results.

At the level of interaction categories, object manipulation and UI activation showed strong associations with general and specific IC as well as prior 3D experience, while spatial navigation appeared less indicative. One plausible explanation is the simplicity and uniformity of the navigation tasks in this study, which relied on basic teleportation in a confined virtual space. In more complex simulations requiring advanced spatial orientation or locomotion under time pressure, navigation may pose high cognitive demands [39,40] and could be more substantially associated with IC or related human abilities.

With regard to the third research objective, both general and specific IC showed moderate, borderline-significant correlations with students’ medical performance as measured by the VR-OSCE station. Beyond the relatively small sample size limiting statistical power, several methodological and contextual factors may have limited the observable correlation between IC and medical performance. First, the original VR-OSCE station allowed for technical assistance from faculty members. Such instructor-mediated compensation strategies can mitigate usability barriers, particularly for users with lower digital competence, as noted in digital learning contexts [41]. However, this likely reduced the influence of IC on medical performance in our setting. Furthermore, the original medical checklist in the VR-OSCE station also included two verbal questions (about the correct diagnosis and the correct sequence of actions), which could be answered without interaction (and thus without engaging IC) and may therefore have weakened the observed correlations. Although the correlations did not reach conventional significance thresholds, the consistent direction and convergence of effects across both types of IC—despite differing operationalizations—support the construct relevance of IC in performance-based VR assessment. This is in line with arguments emphasizing that near-significant effects, particularly when consistent across measures, may still reflect meaningful relationships [42]. The lack of correlation between both types of IC and overall OSCE medical performance suggests that IC is unlikely to be a relevant factor in traditional analog assessment settings. Apparently, IC as a technical, practical skill forms a psychometrically distinct construct from analytical or academic intelligence - a distinction supported by seminal intelligence theories [43]. In line with this, empirical studies have shown that practical intelligence correlates poorly with general cognitive ability, yet predicts job-relevant outcomes, even after controlling for general intelligence [44]. Taken together, our preliminary findings underscore the need for systematic assessment in larger cohorts to better understand the role of IC in VR-based assessment of medical performance: From a psychometric perspective, addressing potential construct-irrelevant variance is essential to uphold the validity and interpretability of performance assessments [19], particularly as immersive technologies are scaled for high-stakes examinations. From an equity standpoint, ignoring individual differences in IC may unintentionally privilege those with prior exposure to similar technologies—such as through gaming—thus reinforcing a second-order digital divide [45]. Therefore, integrating specific IC measures in VR-based examinations may help distinguish true deficits in knowledge and/or practical skills from challenges in VR interaction. This could inform both targeted training interventions and adjustments to assessment design to ensure fairness.

### Limitations

Several limitations of this exploratory study should be noted. First, the sample size was small and relatively homogeneous, which limits the generalizability of the findings. Accordingly, statistical power to detect associations between specific immersive competence and performance was limited, and effect estimates may be unstable or not replicate in a larger sample.

Second, although the checklist for specific IC showed promising internal consistency and item-level characteristics, external validation by independent raters is still pending. Since the measure was assessed using a single domain-specific application (STEP-VR), the generalizability of a checklist-based approach to other VR systems or medical domains also remains to be established.

Third, retrospective recruitment may have introduced selection bias, as participants who volunteered for follow-up testing may have had above-average motivation or technical interest.

Fourth, participants had previously been exposed to the VR-OSCE station and preparatory training. While we assumed that exposure effects had subsided after six months, residual familiarity with the application might still have facilitated specific IC performance, potentially inflating scores. Similarly, frequent interim use of VR could have led to changes in IC since the original performance assessment. However, this appears unlikely considering the low levels of VR exposure reported by participants.

Fifth, specific IC scores may be influenced by system-related factors, such as software constraints or hardware-related issues (eg, controller tracking stability or attachment problems), which were not systematically assessed in this study. While no major technical failures occurred, such factors could have introduced additional variance unrelated to the intended construct.

Sixth, IC may manifest differently depending on the type of immersive system and interaction technique used (eg, controller-based VR, hand-tracking VR, or mixed reality), each relying on distinct interaction metaphors and motor demands. As a result, the present findings are specific to the controller-based VR setup used and may not directly generalize to other immersive systems.

Finally, the correlational design precludes causal conclusions, which future studies could address by manipulating IC (eg, through targeted training) and evaluating its impact on medical performance.

### Conclusions

Despite its limitations, this pilot study provides initial evidence that general and specific IC may be distinct, meaningful, and psychometrically accessible constructs in the context of VR-based medical assessments. The proposed in situ approach—via guided, domain-relevant interaction tasks—demonstrated adequate item characteristics for early-stage evaluation and may provide a practical approach to gauge VR user readiness. While correlations with medical performance in the current sample were limited, this may reflect limited statistical power, ceiling effects, instructor compensation, or the independence of certain verbally addressed OSCE items from IC. Nevertheless, the increasing adoption of immersive technologies in high-stakes training and assessment underscores the potential relevance of IC as a determinant of user performance.

Future research should refine the item design, validate IC measures in larger and more diverse populations, and conduct interventional studies to clarify causal pathways. Such work may help inform strategies to mitigate performance inequities related to operational readiness and potentially enhance the fairness and validity of VR-based assessments in medical education.

## Data Availability

The datasets generated and analyzed during this study are fully provided in the Supplement (Multimedia Appendices 1-3). The VR competence app used in this study was designed by the Chair of Human-Computer-Interaction at the University of Würzburg. Requests regarding its use for research purposes should be directed to the corresponding author. The software STEP-VR (Simulation-based Training of Medical Emergencies for Physicians Using Virtual Reality) used in this study is licensed by ThreeDee GmbH. Requests regarding its use for research purposes should be directed to the company.

## Appendix

Multimedia Appendix 1 Original item set for specific IC.

Multimedia Appendix 2 Significance values for Pearson correlation data from Figure 3 as well as non-parametric Spearman correlation analyses.

Multimedia Appendix 3 Raw data generated and analyzed during the study.

## Abbreviations

IC: immersive competence
OSCE: objective structured clinical examination
STEP-VR: Simulation-based Training of Medical Emergencies for Physicians Using Virtual Reality
TA-EG: Affinity for Technology- Electronic Gadgets
UI: user interface
VR: virtual reality
